# BaShoF: A symbolic dataset of Bach and Shostakovich fugues for polyphonic pattern matching with original, augmented and corrupted queries

**DOI:** 10.1016/j.dib.2026.113339

**Published:** 2026-10-14

**Authors:** Antonio Rizzitiello, Riccardo Giampiccolo, Augusto Sarti, Alberto Bernardini

**Affiliations:** Dipartimento di Elettronica, Informazione e Bioingegneria (DEIB), Politecnico di Milano, Piazza L. Da Vinci 32, Milan, 20133, Italy

**Keywords:** Music Information Retrieval, MIDI corpus, Ground-truth annotation, Query augmentation, Query corruption

## Abstract

This article presents BaShoF (Bach–Shostakovich Fugues), a symbolic dataset of query subjects designed for musical pattern matching, created from the first 12 fugues of J. S. Bach's The Well-Tempered Clavier, Book I, and the first 12 fugues of D. Shostakovich's Op. 87. The dataset includes the 24 original MIDI subjects and a controlled layer of noisy subjects generated from the original ones, with five noise levels, ranging from 10% to 50%, and ten seeds per level. This produced 1200 augmented subjects with added outlier notes and 1200 corrupted subjects with pitch alterations. For each complete MIDI fugue, a CSV file defining a set of overlapping windows is provided together with a window-level ground truth, based on musicological sources, that contains the indices of the windows where subjects occur. In total, the dataset provides 393 ground-truth windows distributed across 24 fugues. All the Python scripts used for window generation and noise addition are included. This dataset can be valuable for testing the accuracy and noise tolerance of pattern-matching systems in polyphonic music.

Specifications Table

**Table:** 

Subject	Computer Sciences
Specific subject area	Music information retrieval and symbolic polyphonic pattern matching.
Type of data	Raw data (MIDI), Processed data (MIDI and CSV), Tables (CSV), Text files (TXT), Code (Python scripts).
Data collection	Original subjects were manually sequenced using MIDI editing software (PreSonus Studio One 5). Augmented and corrupted query subjects were generated using Python (numpy and pretty_midi libraries) starting from the original subjects. Overlapping temporal windows were also generated through a custom Python script to compile ground truth annotations based on musicological sources.
Data source location	Institution: Dipartimento di Elettronica, Informazione e Bioingegneria (DEIB), Politecnico di Milano City/Town/Region: Milan, Lombardy Country: Italy Primary data sources: J. S. Bach's first 12 fugues from The Well-Tempered Clavier, Book I, encoded by David Huron (1994) and obtained from KernScores; D. Shostakovich's first 12 fugues from Op. 87, encoded by José Oscar de Almeida Marques (2002) and obtained from Kunst der Fuge.
Data accessibility	Repository name: Zenodo Data identification number: 10.5281/zenodo.22035056 Direct URL to data: https://doi.org/10.5281/zenodo.22035056
Related research article	A. Rizzitiello, R. Giampiccolo, A. Frigeri, A. Sarti, A. Bernardini, Chamfer and Percentile Hausdorff Distances for Polyphonic Music Pattern Matching, manuscript submitted in co-submission to Pattern Recognition Letters.

## 1 Value of the Data

• The dataset is based on 24 classical contrapuntal works and is, to our knowledge, the first publicly available dataset to offer an operational conversion of existing fugue analyses into a window-indexed benchmark for polyphonic pattern-matching systems. It includes the original subjects used as queries, the CSV files that define the overlapping windows for each fugue, controlled noisy query variants, a window-level ground truth, a URL index for the complete scores, and the Python scripts used for generating the overlapping windows and the noisy queries. The ground truth specifies the windows in which each fugue subject occurs and was constructed through a manual labelling process that associated each subject occurrence with its corresponding window based on musicological sources [[1], [2], [3], [4], [5]], saving researchers extensive annotation effort.
• 2400 variants of the original subjects are included in the dataset, specifically 1200 augmented subjects and 1200 corrupted subjects (in MIDI format). These new fragments, obtained by modifying the original files, have been generated at five increasing levels of noise. The 24 original subjects (in MIDI format), manually sequenced, are also provided.
• Researchers can reuse this dataset to test the accuracy of their polyphonic pattern-matching algorithms and assess their robustness to noise.
• The included Python scripts can also be used to apply the same windowing and noise generation methodologies to other MIDI files.

## 2 Background

The purpose of this dataset is to provide a framework for testing pattern-matching systems in a classical polyphonic context. While there are polyphonic datasets designed to test *pattern discovery* systems [6,7], where input queries are absent, *pattern matching* systems inherently require predefined queries. Existing resources for pattern matching are mainly restricted to monophonic music [8,9], particularly folk songs [10,11], or jazz solos [12], and rarely provide explicitly curated ground truths.

Furthermore, while prior corpora provide formalized ground-truth data for fugues [2], they are designed for symbolic-level musicological analysis without providing a conversion from symbolic subject positions into a window-level pattern-matching ground truth.

Crucially, none of these resources offer the addition of noise in a controlled manner to assess the algorithmic robustness.

The BaShoF (Bach–Shostakovich Fugues) dataset is designed to facilitate pattern-matching experiments using contrapuntal subjects as queries under noise-free and noisy conditions. It is used in the related research article (see supplementary material) to evaluate the performance of the proposed geometric pattern-matching approach. This article adds value by documenting the construction of the corpus, the creation of the window-level ground truth, the generation of augmented and corrupted queries, and the organisation of files and scripts.

## 3 Data Description

The dataset is organised into a root directory containing three Python scripts which are *build_window_indices.py, augment_query.py* and *corrupt_query.py*, three folders, which are *1-original, 2-augmented* and *3-corrupted* and a CSV file containing the URLs of the complete fugue MIDI files. Additional README and license files are also provided.

Given a MIDI file and a window size (in beats), the *build_window_indices.py* script creates an ordered list of overlapping windows and writes a summary CSV file as output. Then, given a MIDI file and a noise level as input, the *augment_query.py* script generates a new MIDI file by adding outlier notes to the original file. In a similar way, the *corrupt_query.py* script creates a new MIDI file through pitch alteration of the original file. Fig. 1 summarizes the main directory structure of the dataset.

**Fig. 1:**
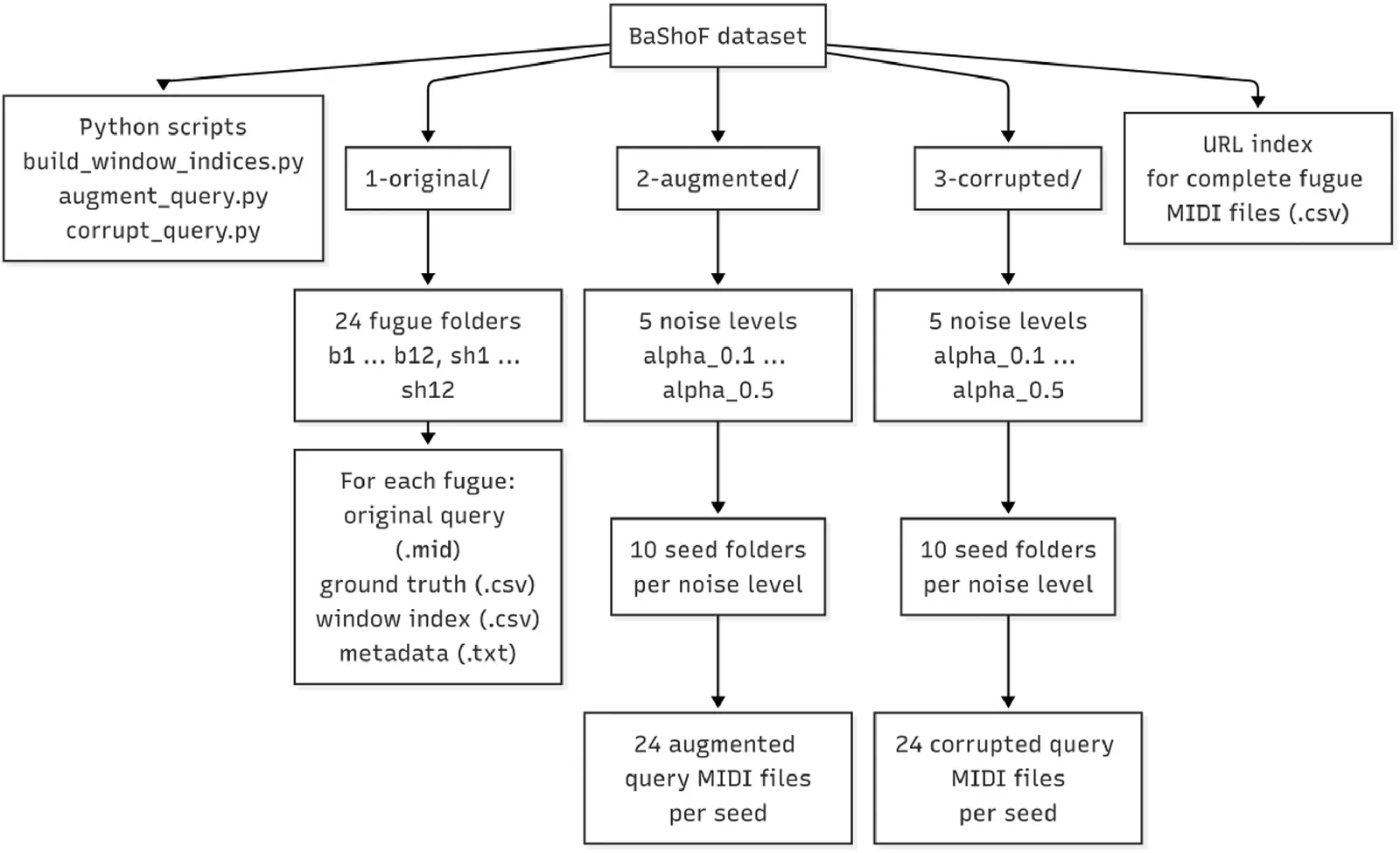
Directory structure of the BaShoF dataset.

The *1-original* folder is divided into 24 subfolders, where each subfolder corresponds to one fugue. The subfolders *b1*-*b12* correspond to Bach's fugues, while *sh1*-*sh12* correspond to Shostakovich's. Each subfolder includes:

• A MIDI file of the original query subject, manually sequenced, related to the reference fugue (e.g., *querybach1.mid*);
• A summary CSV file listing all the windows of the complete fugue (e.g., *wtc1f01_windows_index.csv*);
• A CSV file containing the ground truth for the fugue, represented as an ordered sequence of indices of the windows in which the query subject occurs (e.g., *gt_bach1.csv*);
• A TXT file that includes the metadata and ground truth of the fugue in terms of measures. Here, bar numbers correspond to the original reference scores.

The *2-augmented* folder contains the augmented query subjects obtained by adding extra outlier notes. The folder is divided into five subfolders (e.g., *alpha_0.1* to *alpha_0.5*), each corresponding to a specific increasing noise level. Furthermore, each alpha folder contains 10 subfolders (e.g., *seed_ID* to *seed_ID+10^10*) related to the seeds used for the pseudorandom generation of the augmented queries. Finally, each seed folder contains 24 augmented query subjects, one for each fugue (e.g., *querybach1_aug.mid*). The dataset comprises 1200 augmented subjects.

The third and final folder, *3-corrupted*, has the same hierarchical structure as *2-augmented*, but contains query subjects obtained by pitch alteration rather than by adding outlier notes (e.g., *querybach1_corr.mid*). There are in total 1200 corrupted subjects.

This dataset includes short MIDI excerpts derived from D. Shostakovich's Op. 87. These excerpts are not covered by the dataset's open license and are not placed in the public domain. Rights in the underlying compositions remain with the respective copyright holders.

In this dataset, “beats” refer to quarter-note durations, which are computed based on MIDI Pulses Per Quarter note (PPQ) resolution (see supplementary material). The same ground truth is used for each original query and its corresponding augmented and corrupted variants.

Table 1 summarizes the structure of the dataset.

**Table 1:** Summary metrics for the Bach and Shostakovich fugue dataset.

Metric	Overall Dataset	J. S. Bach Corpus (n = 12)	D. Shostakovich Corpus (n = 12)
Original Queries	24	12	12
Total Notes in Original Queries	414	168	246
Query Length (Notes)	Mean: 17.25 (Range: 5–39)	Mean: 14.0 (Range: 5–26)	Mean: 20.5 (Range: 8–39)
Query Pitch Span	Mean: 11.54 semitones	Mean: 10.08 semitones	Mean: 13.00 semitones
GT Windows	393	189	204
GT Windows per Fugue	Mean: 16.38 (Range: 8–31)	Mean: 15.75 (Range: 8–31)	Mean: 17.0 (Range: 10–26)
GT Window Lengths	Mean: 15.29 beats	Mean: 10.67 beats	Mean: 19.92 beats
Augmented Queries	1200	600	600
Corrupted Queries	1200	600	600

Finally, the root directory also includes a CSV file containing the URLs to download the complete original MIDI files for all 24 Bach and Shostakovich fugues from KernScores and Kunst der Fuge websites (see the next section).

## 4 Experimental Design, Materials and Methods

The dataset was curated using the following sources:

• J. S. Bach's first 12 fugues from The Well-Tempered Clavier (Book 1), encoded by David Huron (1994), obtained from the KernScores website [13].
• D. Shostakovich's first 12 fugues from Op. 87, encoded by José Oscar de Almeida Marques (2002), obtained from the Kunst der Fuge website [14].

These contrapuntal compositions were selected for their relevance to the identification of recurring patterns. They are based on the repetition and transformation of a theme (the initial subject), which allows for a reproducible definition of the ground truth characterized by multiple occurrences (this would be more difficult with repertoires that are less thematically structured). The subject occurrence annotations are converted into window-level labels, which are suitable for pattern matching evaluation. These annotated occurrences include identical entries, transposed entries, incomplete entries, and rhythmic or melodic variations of the original subject. All analysed fugue subjects are monophonic, while the scores are polyphonic. A unique ID is assigned to each fugue: ID 1–12 for Bach's fugues (1–12) and ID 13–24 for Shostakovich's fugues (1–12); this ID is used for seed generation, as explained in subsection 2.

### 4.1 Windowing and preparation of ground truth data

The process of windowing consists of generating overlapping windows from a MIDI file given a predefined window length (see *build_window_indices.py* script). The windows are ordered by the onset time of their lead note, and for simultaneous notes (i.e., a chord), a separate window is generated for each note in the chord, with each serving as the lead note. In our dataset, the length of the windows varies for each fugue, ensuring that the subject is contained in its entirety (with a small variable margin). The exact window length for each fugue is defined in the respective metadata TXT file. These lengths reproduce the benchmark windowing configuration used in the related study (see supplementary material); users may generate alternative window lengths with the supplied *build_window_indices.py* script.

The script outputs a CSV file listing all windows by index, start and end times (in beats), as well as their lead and end pitches (MIDI pitch numbers). Based on this list of indices, all windows corresponding to an occurrence of the subject were selected for each fugue, producing the window-level ground truth. Various analyses from the literature were used to identify subject positions in each fugue for both Bach [[1], [2], [3]] and Shostakovich [2,4,5], and following the initial setup, a second annotator independently verified all mappings.

In particular, we consider a window relevant only if it contains a subject occurrence from the musicological source and their lead-note start times coincide (from now on referred to as “onset-aligned”). Windows that only partially overlap a subject occurrence are treated as non-relevant. Therefore, an algorithm's performance can be negatively impacted if partial overlaps are ranked higher than, or among, the relevant windows; if all relevant windows are ranked above them, the evaluation is unaffected.

We opted for this criterion as it allows each occurrence of the subject to be unambiguously associated with the corresponding window, and is therefore more stringent than a simple partial overlap. The rationale behind this choice is that the identification of an onset-aligned window corresponding to an occurrence usually represents a more precise match than the identification of a window that captures only part of it. For this reason, we consider onset-aligned windows as a valid benchmark for testing a pattern-matching algorithm, even though the ground truth does not classify partially overlapping windows as relevant.

In Fig. 2, we show the original query subject used for Bach's Fugue No 2 in C minor from The Well-Tempered Clavier, Book I, and in Fig. 3, the first 21 bars of this fugue along with the first six subject occurrences appearing in this excerpt (which are included in the ground truth). The query subject's notes are highlighted in dark blue, while the fugue notes are shown in grey. Subject notes and score notes overlap where an occurrence is present.

**Fig. 2:**
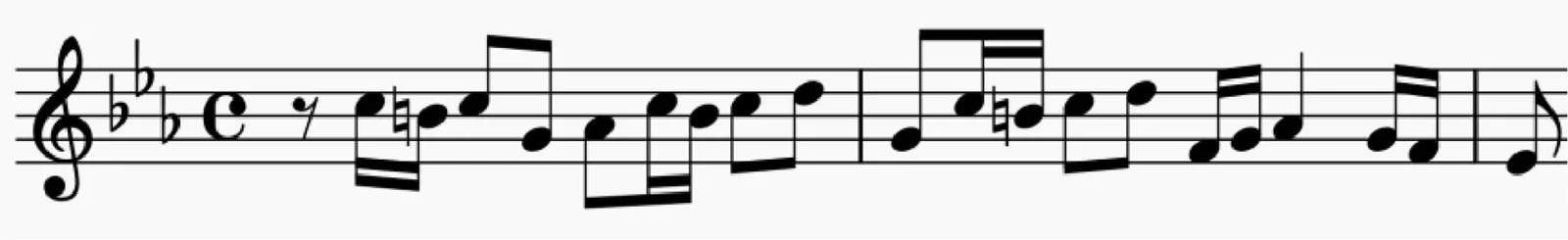
Musical notation of the original query subject from Bach's Fugue No 2 in C minor, BWV 847 (based on [1]).

**Fig. 3:**
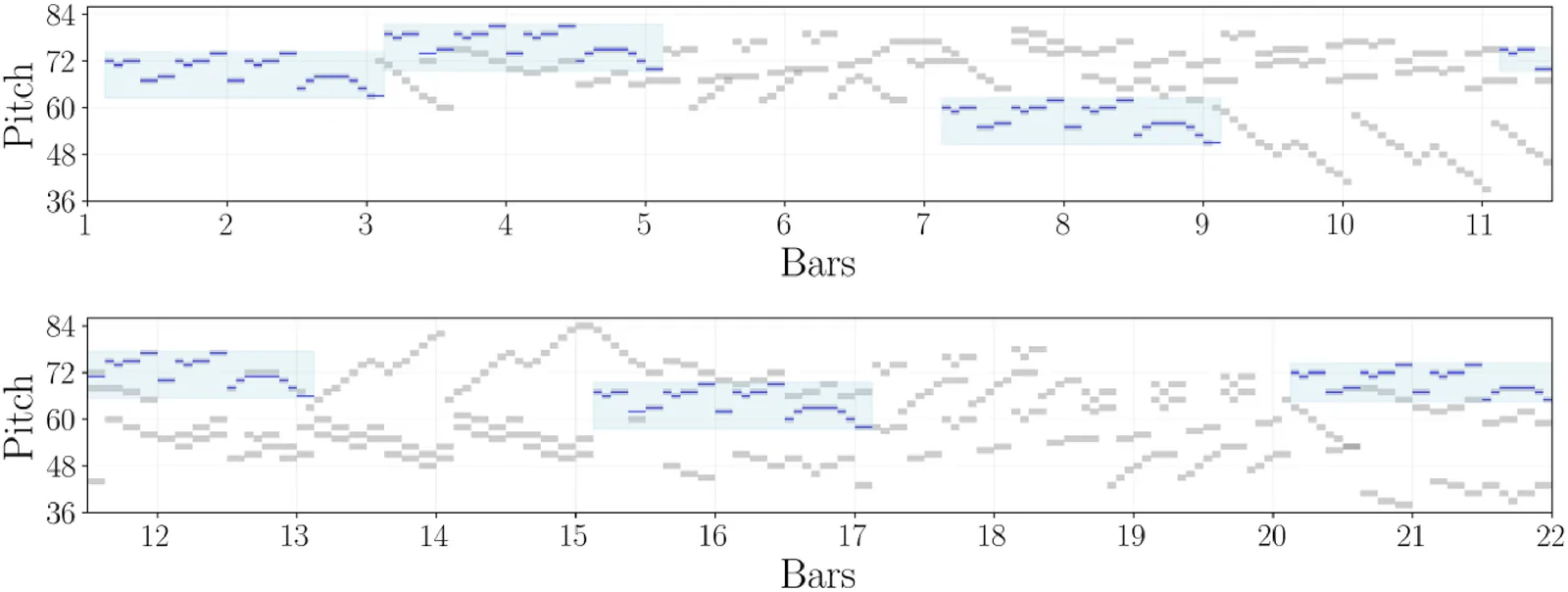
First 21 bars of Bach's Fugue No 2 in C minor, and the first 6 subject occurrences.

### 4.2 Noisy queries

This dataset enables researchers to test the robustness of their pattern-matching algorithms by providing a large corpus of noisy queries derived from the original set. The initial 24 subjects were modified to generate 1200 augmented and 1200 corrupted queries, which are distributed across five different noise levels *α* (with *α* ranging from 0.1 to 0.5). Each level represents the nominal fraction of notes added or modified relative to the original subject. For a query with *m* notes and a noise level *α*, the number of added or modified notes, *k*, is given by *k =* ⌈*αm*⌉. For example, to alter 20% of the notes in a fragment of 10 notes (i.e., *α* = 0.2 and *m* = 10), applying this formula gives *k* = 2. The ceiling function is applied to always return an integer number of notes and ensure that at least one note is altered even for short queries or small values of *α*.

In particular, for each level of noise, the 24 subjects were altered pseudorandomly using 10 deterministically defined seeds to produce 10 variants, and this was done to reduce dependence on a single pseudorandom realization. In other words, the selection of notes to be altered was repeated 10 times for each fugue and for each noise level, using a different seed for each of the 10 iterations. Overall, this corresponds to 10 seeds × 24 fugues × (5 levels of augmentation + 5 levels of pitch corruption), making a total of 2400 altered queries.

The seeds are defined deterministically as follows: the *fugue ID* is used as the random seed in the first iteration. From the second to the tenth iteration, the seeds are obtained by adding the *fugue ID* to a power of 10 (e.g., *fugue ID+10², fugue ID+10³*, and so on up to *fugue ID+10¹⁰*).

The selection of notes to be altered was carried out using the same uniform sampling rules for all queries within each perturbation type and at each noise level; therefore, the noise is generated according to a consistent stochastic procedure.

In our approach, we chose five noise levels (i.e., *α* ∈ {0.1,0.2,0.3,0.4,0.5}) for the following reasons: first, they are evenly spaced and represent a gradual increase in alteration (from mild to substantial). This allows us to check how performance evolves as noise increases, while ensuring a balanced representation of the degradation intervals. Second, imposing a maximum of 50% allows us to preserve a meaningful portion of the original query during the stress test, enabling us to effectively test the limits of an algorithm without excessively altering the original query with too much noise.

The augmentation and corruption processes are carried out as follows:

• **Query augmentation** (see *augment_query.py*). This script adds *k* outlier notes to an original MIDI file. For each outlier, a reference note is randomly selected with a uniform distribution from those that do not belong to the initial onset of the fragment (in this way, the initial onset of the subject, which in some systems can serve as an anchor [15], remains unchanged). The onset time and duration of the new note coincide with those of the reference note, while the pitch is obtained by adding to the base pitch an integer delta uniformly drawn from {−12,…,−1,+1,…,+12}. New notes that would produce duplicate pitch-onsets or pitches outside the MIDI range are discarded and regenerated.
• **Query corruption** (see *corrupt_query.py*). This process functions identically to the augmentation process, except that instead of generating outliers, it modifies the pitch of *k* distinct existing notes that do not belong to the initial onset by an integer delta (uniformly drawn from {−12,…,−1,+1,…,+12}). As in the previous case, any notes resulting in duplicate pitch-onsets or pitches outside the MIDI range are discarded and regenerated.

These perturbations are primarily intended as a tool for carrying out controlled stress tests in the context of pattern matching, rather than an attempt to generate stylistically or musically plausible variations. The augmentation/corruption process, as defined, therefore produces MIDI sequences that can be harmonically and contrapuntally imperfect.

In real-world scenarios, augmentation could be interpreted as the erroneous and unintentional insertion of notes not present in the original score, while pitch alteration acts as a substitution error. These errors could occur, for example, if a user searches for a musical fragment within a score database but enters an imprecise query that does not perfectly match the fragment they wish to find (simply because the notes may not be remembered correctly), or if the given query comes from a poor transcription or even a live performance. Consequently, it is useful to stress-test a pattern-matching algorithm using BaShoF to determine whether it can perform well despite noisy queries.

Fig. 4 shows the piano-roll representation of the original query subject from Bach's FugueNo. 2 in C minor; Fig. 5 shows its augmented version, and Fig. 6 its corrupted variant. In this example, both the augmented and corrupted sequences are obtained by applying a noise level *α* = 0.1 (meaning 2 added/corrupted notes out of 20, specifically D3 and A3 outlined in red), and a random seed of 102, obtained by adding the *fugue ID* (2) and 10².

**Fig. 4:**
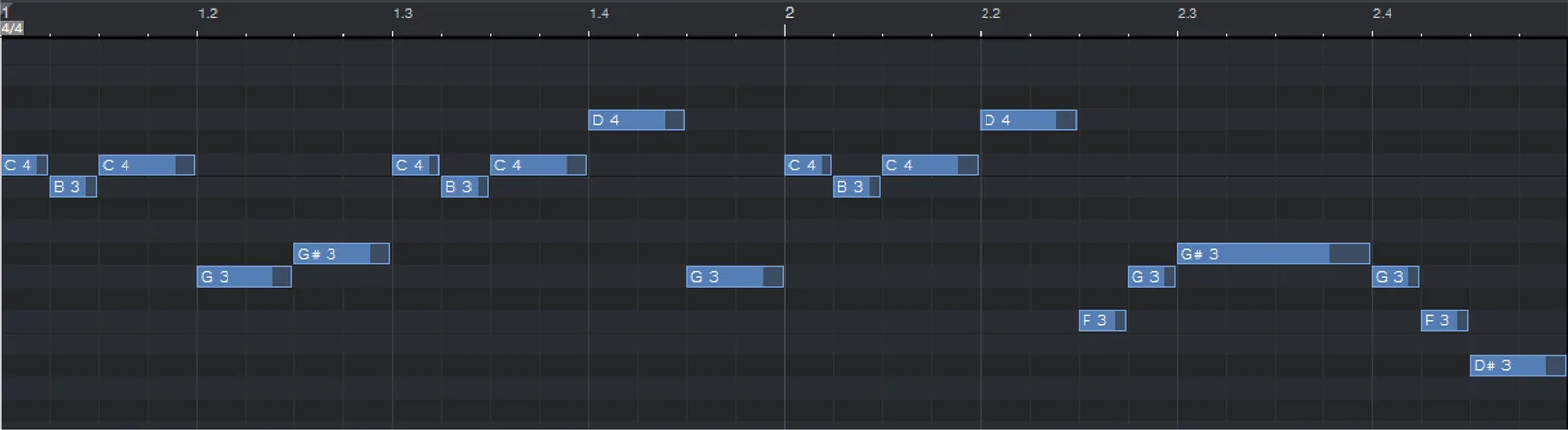
Piano-roll representation of the original query subject from Bach's Fugue No. 2 in C minor, BWV 847, visualized in PreSonus Studio One 5.

**Fig. 5:**
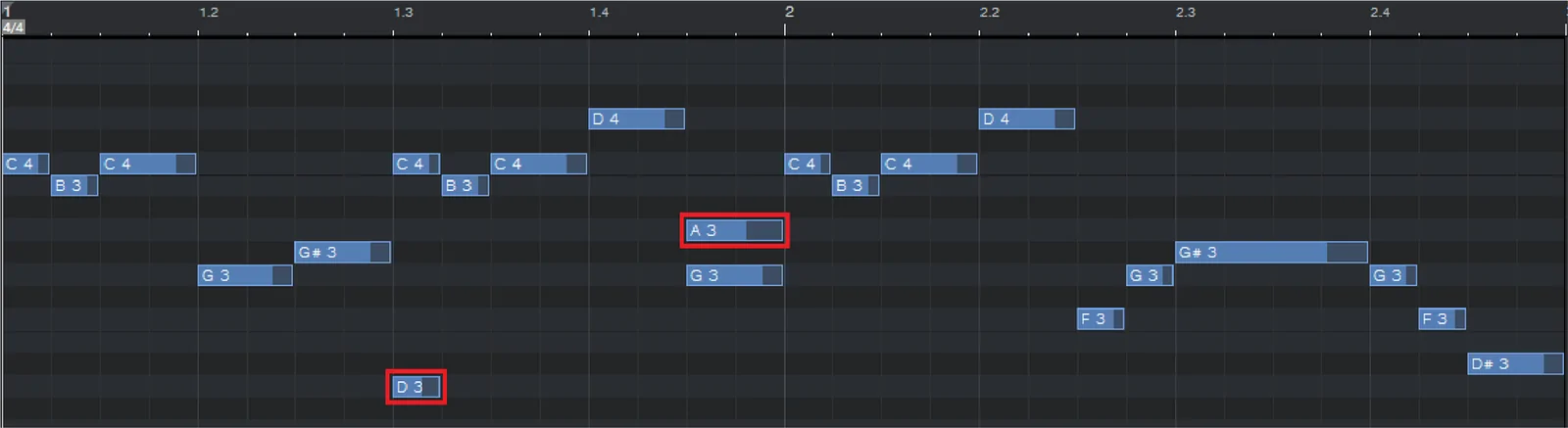
Augmented version of the query subject shown in Fig. 4, generated with noise level *α* = 0.1 and random seed 102. Added notes are outlined in red.

**Fig. 6:**
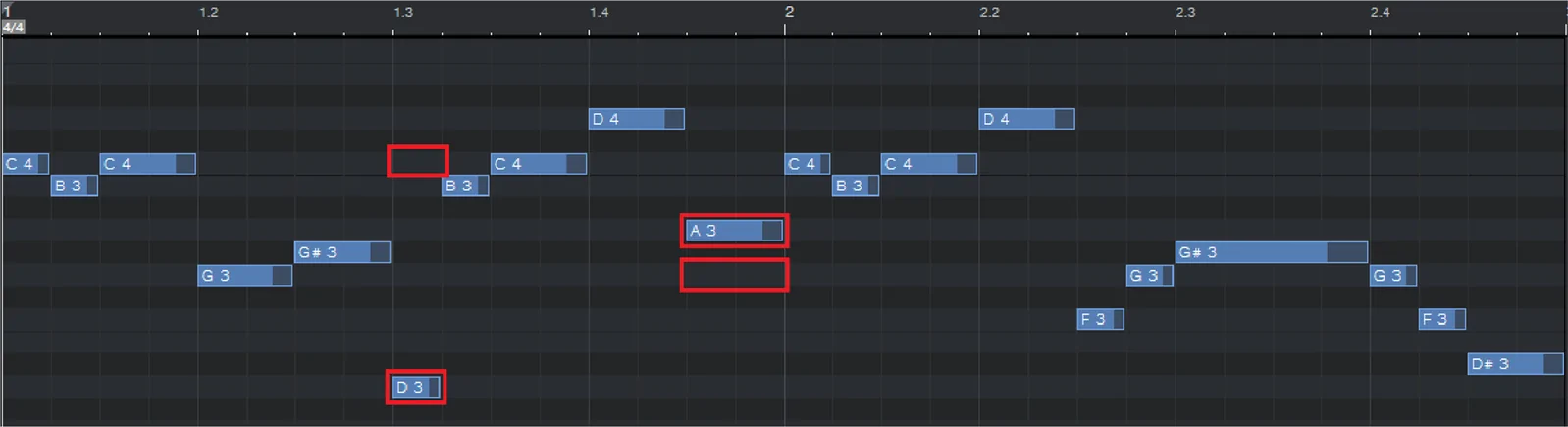
Corrupted version of the query subject shown in Fig. 4, generated with noise level *α* = 0.1 and random seed 102. For each corrupted note, the original note position is indicated by an empty red outline, while the replacement note is outlined in red.

All query subjects (original, augmented and corrupted) are onset-normalized so that the first note starts at time zero.

Although this dataset is designed to test pattern-matching algorithms, it could also be adapted for pattern discovery, in order to assess how effectively the subject of each fugue can be identified from its occurrences without providing any input query. However, it is important to note that, first, occurrences of the subject are not only shifted in time and pitch, but are often musically varied or transformed with respect to the original subject. This may be problematic for algorithms designed to detect repeated patterns only when differences are limited. Second, the ground truth provided in this dataset is restricted to subject occurrences and does not include all repeated patterns within each piece. These aspects should therefore be taken into account when evaluating a pattern-discovery algorithm.

## Limitations

One of the main limitations of this dataset is that it is based exclusively on Western classical music. Bach's fugues, as well as those of Shostakovich, are characterised by musical properties that are specific to a particular contrapuntal style. The dataset does not include pieces from other musical traditions and genres, such as non-Western repertoires, jazz or popular music, in which characteristics such as rhythmic and pitch organisation can differ significantly. This inherently produces a bias towards Western classical music.

Another limitation is that the ground truth is annotated at the window level rather than the note level. That is, the ground truth is given by the set of windows where an instance of the query subject exists, without specifying the exact notes that constitute that occurrence. This design favours algorithms that operate on time windows; conversely, note-based algorithms must account for this aspect when processing the ground truth.

Finally, it should be noted that augmented and corrupted subjects were generated from the original subjects via an algorithmic process. While the resulting subjects ensure the possibility of conducting a useful stress test of pattern-matching systems, they do not necessarily reproduce the imperfections of human performance.

## Ethics Statement

The authors have read and followed the ethical requirements for publication in Data in Brief and confirm that the current work does not involve human subjects, animal experiments, or any data collected from social media platforms.

## CRediT Author Statement

**Antonio Rizzitiello:** Writing - Original Draft, Investigation, Conceptualization, Methodology, Resources, Software, Formal Analysis, Validation, Data Curation; **Riccardo Giampiccolo:** Writing - Review & Editing, Formal Analysis, Methodology, Supervision, Project Administration; **Augusto Sarti:** Writing - Review & Editing, Supervision, Project Administration; **Alberto Bernardini:** Writing - Review & Editing, Supervision, Methodology, Project Administration.

## Data Availability

BaShoF: A Symbolic Dataset of Bach and Shostakovich Fugues for Polyphonic Pattern Matching with Original, Augmented and Corrupted Queries (Original data).

## Appendix Supplementary materials

Image, application 1
