# Supplementary material for: BaShoF: A symbolic dataset of Bach and Shostakovich fugues for polyphonic pattern matching with original, augmented and corrupted queries
Source: Data Brief. 2026 Oct 14;69:113339. doi: 10.1016/j.dib.2026.113339 (PMC13695106; doi:10.1016/j.dib.2026.113339)
Supplement: Supplementary file 1 [file mmc1.pdf]

Chamfer and Percentile Hausdorff Distances for Polyphonic Music Pattern Matching

Antonio Rizzitiello[a], Riccardo Giampiccolo[a,*], Achille Frigeri[a], Augusto Sarti[a] and Alberto Bernardini[a]

[a]*Dipartimento di Elettronica, Informazione e Bioingegneria (DEIB), Politecnico di Milano, Piazza L. Da Vinci 32, Milan, 20133, Italy*

ARTICLE INFO

Keywords:
Music Information Retrieval
Pattern Matching
Chamfer Distance
Hausdorff Distance

ABSTRACT

Pattern matching in symbolic music represents a challenging retrieval problem in which a short fragment must be identified within a larger score. In this context, an established geometric technique is the directed Hausdorff distance, which evaluates musical similarity by considering the maximum nearest-neighbor distance between two point sets. In this article, we propose a more robust geometric approach utilizing three families of distances: the directed Chamfer distance, the trimmed Chamfer distance, and the percentile Hausdorff distance. This approach is tested using BaShoF, a polyphonic pattern-matching dataset created from the first twelve fugues of J. S. Bach's The Well-Tempered Clavier, Book I and D. Shostakovich's Op. 87. Experiments are conducted using the original queries from the dataset, as well as augmented and corrupted queries with five levels of added noise ranging from 10% to 50%. Mean Average Precision is used to measure retrieval performance. Our results show that the proposed alternatives outperform the directed Hausdorff baseline in retrieval accuracy, with the directed Chamfer performing best on original queries, and trimmed Chamfer being superior against noise caused by both added outlier notes and pitch corruption.

1. Introduction

Within the field of Music Information Retrieval (MIR), a key challenge lies in defining a notion of similarity that is both musically valid and computationally feasible [1]. This challenge is central to cataloguing, classification, and musicological analysis, and is known to be particularly tied to the choice of musical representation [2]. Addressing it requires taking into account multiple perceptual attributes that listeners use to identify matches between musical fragments. For instance, empirical studies indicate that listeners recognize melodies across pitch transpositions [3], perceive ornamental notes as expressive embellishments [4], and accept minor temporal variations as natural expressive nuances [5].

For the evaluation of similarity in symbolic music, the main approaches include geometric [6–10], string-matching [11–13], n-gram [14, 15], tree-based [16], graph-based [17], and probabilistic methods [18], as well as recent techniques based on neural networks [19, 20]. In this article, we focus on geometric approaches, which commonly model notes as points or horizontal segments, and prove to be particularly effective for the analysis of polyphonic scores given their multidimensional nature. In particular, one key geometric method is based on the application of the Hausdorff distance [21], whose goal is to quantify how much two sets of points differ considering the maximum distance of their respective nearest neighbors. Di Lorenzo and Di Maio adapted this concept for the evaluation of monophonic music similarity [8], and Romming and Selfridge-Field later applied the directed variant of the Hausdorff distance for polyphonic music retrieval [10]. Romming's algorithm matches a given query against a full score with the aim of minimizing the directed Hausdorff distance under translation. While this method proves effective at capturing spatial proximity between two sets of points, it is based on a worst-case criterion. Consequently, a single displaced point can dominate the evaluation and mask valid correspondences; in music, for example, one note can easily be displaced during arpeggiation or variation. To address this challenge, we propose a more robust approach articulated in the following contributions:

- We propose a geometric method for polyphonic pattern matching, aimed at locating a pattern inside a larger score, replacing the worst-case behaviour of the classical directed Hausdorff distance with the directed Chamfer [22], trimmed Chamfer, and percentile Hausdorff distances [23]. An input query is matched against candidate windows extracted from the score, which are ranked by increasing distance.
- We implement a directed Hausdorff baseline based on the pattern-matching scheme by Romming and Selfridge-Field [10]. Unlike the $(1+\epsilon)$ nearest-neighbor approximation search associated with Balanced Box-Decomposition (BBD) trees in [10], our implementation computes nearest-neighbor distances exactly to ensure a rigorous evaluation.
- To test our approach, we introduce BaShoF [24], a polyphonic pattern-matching dataset created from the first 12 fugues of J. S. Bach's The Well-Tempered Clavier, Book I, and the first 12 fugues of D. Shostakovich's Op. 87. The dataset includes: the 24 original MIDI subjects (used as queries); a window-level ground truth, based on

*Corresponding author

antonio.rizzitiello@mail.polimi.it (A. Rizzitiello); riccardo.giampiccolo@polimi.it (R. Giampiccolo); achille.frigeri@polimi.it (A. Frigeri); augusto.sarti@polimi.it (A. Sarti); alberto.bernardini@polimi.it (A. Bernardini)

ORCID(s): 0009-0003-4560-852X (A. Rizzitiello); 0000-0001-6144-8288 (R. Giampiccolo); 0000-0002-5062-5303 (A. Frigeri); 0000-0002-5803-1702 (A. Sarti); 0000-0001-7973-0134 (A. Bernardini)

musicological sources, that contains the indices of the windows where subjects appear in each fugue; and 2400 controlled variants of the original subjects for robustness tests. These variants are distributed across five levels of noise (ranging from 10% to 50%) and consist of 1200 augmented subjects with added outlier notes, and 1200 corrupted subjects with modified pitches. Both original and altered subjects can be used for retrieval tasks within each fugue. Finally, a URL index for acquiring the complete fugue MIDI files is also provided.
- The performance is evaluated on original, augmented, and corrupted queries, across six temporal scaling factors. Non-interpolated Average Precision (AP) is used as the main evaluation metric.

To our knowledge, these robust geometric distances have not been applied to symbolic music retrieval. Experiments on the BaShoF dataset show significantly improved performance on original, augmented, and corrupted queries over the classical directed Hausdorff baseline, and, in particular, directed Chamfer maximizes retrieval accuracy on original queries, while trimmed Chamfer proves superior at mitigating added outlier notes and pitch corruption, making both approaches promising for robust motif search in symbolic polyphonic music.

2. Related works

Several approaches have been suggested for the symbolic evaluation of musical similarity. String-based methods compare sequences of symbols using alignment algorithms [11–13]. In these approaches, one key challenge is defining how to encode musical information while preserving both the vertical and horizontal dimensions [25]; for monophonic music (i.e., characterized by a single melodic line), where notes are already ordered temporally, it is possible to encode them sequentially using their pitches or intervals. Instead, in the context of polyphonic music, specific techniques (such as non-interleaved strings, interleaved strings or onset-based strings [26]) are needed in order to linearize musical events.

Approaches based on n-grams, on the other hand, exploit the analogy with natural language to create and compare tokens consisting of contiguous sequences of symbols [14, 15]. Recent approaches explore dynamic n-gram fingerprinting to speed up searches [27], as well as the detection of melodic plagiarism in symbolic data [28].

However, encoding methods based on the use of strings and n-grams may be limited in their ability to represent the structure of musical content. For this reason, many techniques in the literature exploit different representations, including tree-based models that organize music hierarchically, as well as graph-based and probabilistic models that encode tonal and rhythmic relationships [16–18].

In recent years, symbolic music similarity and pattern discovery have moved beyond traditional techniques based on heuristic rules, to focus increasingly on learned representations and deep neural networks [19, 20, 29]. While deep learning models excel at extracting high-level features, such as melodic contour, harmony and style, they require large datasets and often offer limited interpretability [30].

For this reason, geometric methods still hold significant value, as they guarantee deterministic, fully transparent and training-free pattern matching. These approaches represent music as points or horizontal segments in n-dimensional spaces, and evaluate symbolic similarity through geometric concepts such as distances, shapes, or areas [6, 7, 9]. The Hausdorff distance, introduced by Felix Hausdorff [21, pp. 290–294], is one of the key measures underlying the geometric approach by Di Lorenzo and Di Maio [8]. In their approach, the authors formalize a melodic space in which melodies are represented as sets of points defined by time and pitch coordinates. This representation naturally incorporates fundamental properties such as transposition and time-shift invariance, enabling a meaningful assessment of melodic similarity that is consistent with human perception.

Later, this idea was adapted by Romming and Selfridge-Field [10], who extended the application domain to polyphonic music retrieval. In their work, they address the problem of locating a query fragment within a larger score by taking into account the musical invariances previously explored by Di Lorenzo and Di Maio, and formalize it as finding the optimal translation vector that minimizes the directed Hausdorff distance between the query and the score. Romming and Selfridge-Field laid the fundamental groundwork for polyphonic retrieval utilizing the directed Hausdorff distance, providing an efficient approximation algorithm that solves this problem with an $O(mn)$ query time and $O(n)$ space (optimized with BBD-trees and windowing operations). Because the directed Hausdorff metric relies strictly on a maximum nearest-neighbor distance (a worst-case criterion), a single unmatched or spurious note in the query, such as an expressive variation, an ornamental grace note, or an input error, dominates the overall distance calculation. This single outlier can drastically inflate the dissimilarity score and mask true pattern occurrences, motivating the development of robust geometric distance alternatives.

3. Method

This section details the proposed geometric pattern matching framework. First, we formalize the polyphonic retrieval problem. Next, we describe the 3D note representation, the windowing strategy, and the geometric distance functions. Figure 1 summarizes the overall framework.

3.1. Problem Formulation

We formulate symbolic polyphonic pattern matching as a directed retrieval problem. Let S denote a target polyphonic score and $Q = \{q_1, \dots, q_m\}$ a query pattern consisting of m note events. The score is decomposed into a set of n candidate overlapping windows $\mathcal{W} = \{W_1, \dots, W_n\}$, where each W_j, $j = 1, \dots, n$, is a polyphonic fragment of the score, generated according to the windowing process described in Section 3.3. Both the query Q and candidate windows W_j are modeled as point sets in a continuous 3D coordinate space, whose coordinates are defined in Section 3.2. Our goal is to

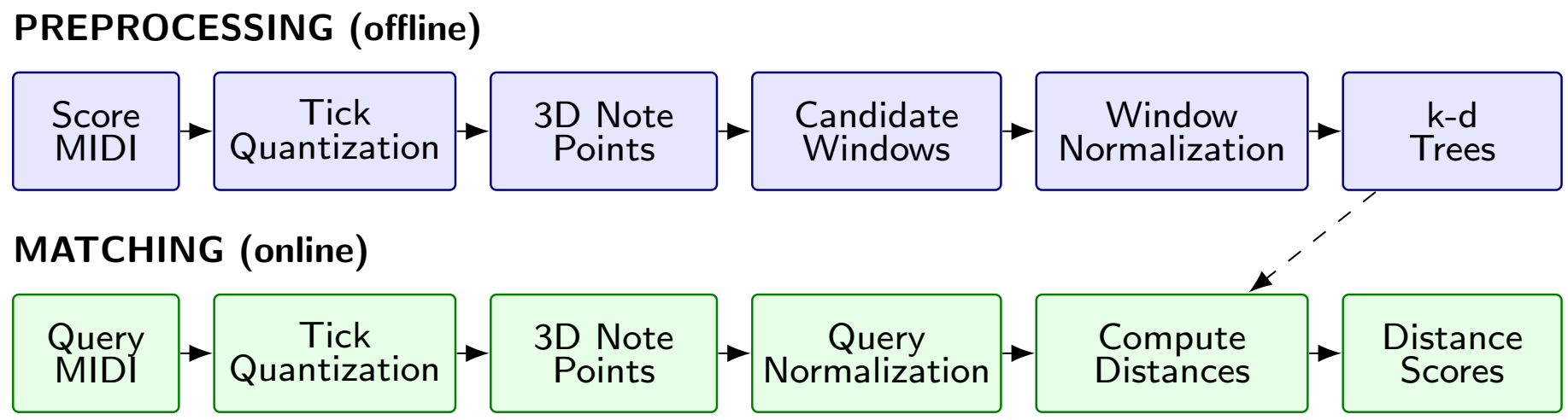

Figure 1: Block diagram of the proposed preprocessing and matching procedure.

identify which windows of S correspond best to Q. In our experiments, S is a complete fugue and Q is the recurrent fugue subject.

A distance function $\mathrm{D}(Q, W_j)$ is used to quantify the dissimilarity between the query and each candidate window. Then, the entire set is sorted from lowest to highest dissimilarity, yielding a permutation $\pi = (\pi_1, \ldots, \pi_n)$, such that

$$\mathrm{D}(Q, W_{\pi_1}) \leq \mathrm{D}(Q, W_{\pi_2}) \leq \cdots \leq \mathrm{D}(Q, W_{\pi_n}).$$

The indices of the manually annotated query occurrences form our ground-truth set [24], denoted as $G \subseteq \{1, \ldots, n\}$. A window is considered relevant if it belongs to G. The effectiveness of a distance function is measured by how highly it ranks the windows in G.

3.2. Representation

Musical information is represented in 3D using pitch p_i, onset time t_i, and duration d_i extracted from the input MIDI file for each note i. Onset times and durations are expressed in quarter-note units by dividing MIDI ticks by the file PPQ (Pulses Per Quarter note). Onset and offset times are tick-quantized with a half-tick tolerance. We express each note as a point $x_i = (p_i, \sigma t_i, \ell_i)$, where $\sigma > 0$ is a scaling parameter, used to control the weight of the onset time coordinate, and $\ell_i = \log_2(d_i/4)$ is the log-duration. This logarithmic scale is used to map equal duration ratios to equal steps on the duration axis (e.g., the distance between a half and a quarter note is identical to the distance between a quarter and an eighth note). Finally, the query and candidate windows are normalised with respect to an anchor note. Given an anchor $a = (p_a, \sigma t_a, \ell_a)$, each note point is shifted to $\tilde{x}_i = (p_i - p_a, \sigma(t_i - t_a), \ell_i)$.

The query anchor is defined as the earliest note, with the lowest-pitched note chosen by default if multiple notes occur simultaneously at the beginning of the query. The anchors of the candidate windows are also chosen among the earliest notes of each window, and this is described in detail in the windowing Section 3.3. This normalization strategy yields transposition and time-shift invariance, enabling direct comparison of transposed and time-shifted occurrences of the same fragment within a common relative representation while preserving the time intervals between notes and their durations.

3.3. Windowing

The windowing process involves generating n overlapping time windows of fixed duration from the analyzed score during the preprocessing phase. After the tick-quantization step, the note events in the score S are organised into ordered groups based on their common onset time t_g. Next, a window is generated from each onset time t_g that spans a time interval of $[t_g, t_g + L)$, where L is the input window length, chosen conservatively to be greater than the query length (the length L used in our experiments for each fugue is specified in the data associated with this study). The window includes all the notes of the score whose onset time falls within this interval.

When several notes start at t_g, each of them is considered in turn as a possible lead note for a window. Thus, each ordered group can generate multiple windows with the same length, but with different anchor notes. Each generated window is normalized by translating it relative to its lead note a, which serves as an anchor, as described in Section 3.2, producing a set of overlapping normalized windows W_j expressed in local coordinates. Finally, each normalized window is stored as a k-d tree, which is used for an efficient nearest-neighbor search during the distance computation phase described in Section 3.4.

3.4. Distance Functions

Given a normalized query $Q = \{q_1, \ldots, q_m\}$ and a normalized candidate window W, we first compute the directed nearest-neighbor discrepancies

$$\delta_i(Q, W) = \min_{w \in W} \|q_i - w\|_2 , \qquad i = 1, \ldots, m ,$$

where w represents a point in W, and $\|\cdot\|_2$ denotes the Euclidean norm.

In our experiments, nearest neighbors are computed exactly (i.e., without approximation techniques). Classical directed Chamfer distance [22] is defined as the average discrepancy

$$\mathrm{CD}(Q, W) = \frac{1}{m} \sum_{i=1}^{m} \delta_i(Q, W) ,$$

whereas classical directed Hausdorff distance [23] is defined by the maximum discrepancy

$$\mathrm{HD}(Q, W) = \max_{1 \leq i \leq m} \delta_i(Q, W) .$$

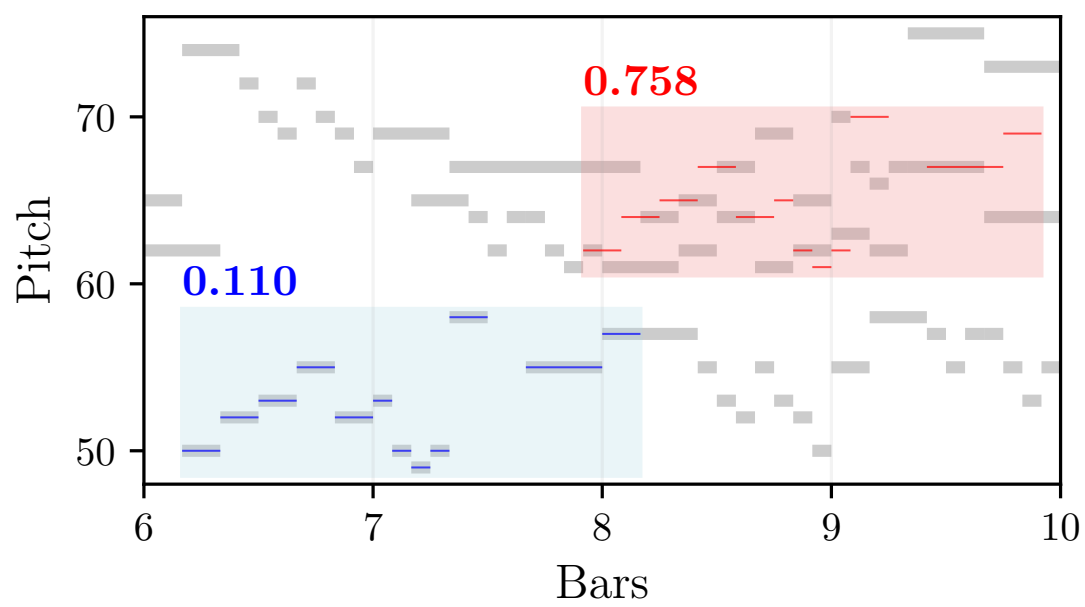

Figure 2: Piano-roll visualization of directed Chamfer retrieval of the fugue subject (Bach WTC I, Fugue 6). The blue window is a ground-truth occurrence, whereas the red window is non-relevant; numbers indicate directed Chamfer distances.

The latter is determined by the worst-matching query event and is therefore sensitive to outliers.

We also consider robust variants to reduce this sensitivity. For fixed Q and W, let $\delta_{(1)} \leq \delta_{(2)} \leq \cdots \leq \delta_{(m)}$ be the sorted nearest-neighbor discrepancies (i.e., order statistics), and let $k_\rho = \max(1, \lfloor \rho m \rfloor)$, with $\rho \in \{0.5, 0.6, 0.7, 0.8, 0.9\}$. The percentile Hausdorff distance is defined as

$$\mathrm{HD}_\rho(Q, W) = \delta_{(k_\rho)},$$

while the trimmed Chamfer distance is defined as

$$\mathrm{CD}_\rho(Q, W) = \frac{1}{k_\rho} \sum_{i=1}^{k_\rho} \delta_{(i)}.$$

Both variants ignore the largest query-to-window discrepancies. Percentile Hausdorff replaces the maximum by an order statistic, whereas trimmed Chamfer averages only the best-matching fraction of query events. After the matching phase, all candidate windows are ranked by increasing distance. Figure 2 shows an example of directed Chamfer retrieval.

3.5. Complexity Analysis

During the matching phase, comparing a given query with all candidate windows requires mn nearest-neighbor operations, where m is the number of notes in the query and n is the number of candidate windows in the score. Since the windowing procedure generates one anchored window for each score note, n is also the number of score notes. By implementing a k-d tree for each window, this comparison can be achieved in average-case $O(mn \log s)$ time for a fixed value of σ, where s denotes the maximum number of notes in a candidate window. If we assume s to be a constant independent of n, meaning that each window contains a bounded number of notes, the complexity becomes $O(mn)$. This asymptotic bound holds for the trimmed and percentile variants as well, as they only introduce an additional $O(m)$ selection step per window. Under the same assumption, the space complexity required to store n k-d trees reduces from $O(ns)$ to $O(n)$. Producing the final ranking of all windows, which is necessary for our evaluation metrics, requires an additional $O(n \log n)$ time. Returning the single best-matching window would reduce this final step to $O(n)$.

During preprocessing, all score notes are read and sorted by onset time in $O(n \log n)$ time, while candidate windows are built and normalized in $O(ns)$ time. Finally, a k-d tree is constructed for each window in $O(ns \log s)$ time. If we treat s as a bounded constant, the overall preprocessing time complexity simplifies to $O(n \log n)$.

4. Experiments

This section outlines our experiments, analyzes the performance of the proposed approach under varying levels of noise, and discusses the obtained results.

4.1. Experimental Setup

For our experiments, we propose BaShoF [24], a dataset of symbolic polyphonic music designed for pattern-matching tests, created from the first 12 fugues of Bach's The Well-Tempered Clavier, Book I, and the first 12 fugues of Shostakovich's Op. 87. The selected works are intrinsically based on the repetition and variation of a theme; this provided an objective and extensive ground truth (with numerous instances to be identified), enabling a robust evaluation of performance that would have been difficult to achieve with less thematically structured repertoires. Ground truth is established based on musicological sources [31, 32], to identify all temporal windows within each MIDI fugue (the target score) that contain occurrences of the primary subject (the query). Retrieval is evaluated at the window level using the annotated windows.

We conduct our tests under three experimental conditions: (i) noise-free, using the 24 subjects as queries in their respective fugues; (ii) augmented, adding random outlier notes within ±12 semitones to the original queries; and (iii) corrupted, altering the pitch of a random subset of notes from the original subjects (also restricted to ±12 semitones). For the final two conditions, we test our approach across five levels of noise α, with $\alpha \in \{0.1, 0.2, 0.3, 0.4, 0.5\}$, representing the nominal fraction of noisy notes introduced with respect to the original queries. By applying ten random seeds to each fugue at every noise level, we generate 240 queries per α level, producing a test set of 1200 augmented queries and 1200 corrupted queries.

We implement our model in Python and perform MIDI parsing and tick conversion using PrettyMIDI. Furthermore, we store each window's point set in a k-d tree using SciPy in order to enable fast nearest-neighbor queries.

The code associated with this study is available at `https://github.com/polimi-ispl/sm_geompm`, the results and overlay plots at `https://zenodo.org/records/22181155`, and the BaShoF dataset at `https://zenodo.org/records/22035056`.

4.2. Evaluation Metrics and Baselines

To obtain a range-agnostic evaluation, we formulate the retrieval problem as a classification task, and use non-interpolated AP to quantify retrieval effectiveness. This metric is computed across unique score thresholds. After assigning a dissimilarity value to each candidate window, $\mathrm{D}_j = \mathrm{D}(Q, W_j)$, we define the retrieval score as $s_j = -\mathrm{D}_j$. Then,

let the distinct ordered values from the set $\{s_1, \dots, s_n\}$ be represented by $\tau_1 > \tau_2 > \cdots > \tau_K$; given a threshold τ_k, we can determine all the windows for which $s_j \geq \tau_k$ and compute the respective precision, P_k, and recall, R_k.

The overall AP is then evaluated as

$$\mathrm{AP} = \sum_{k=1}^{K} (R_k - R_{k-1}) P_k \, , \qquad R_0 = 0 \, .$$

Windows with identical scores are evaluated jointly at the same threshold, avoiding the need to break ties based on the ground truth. Finally, we derive the Mean Average Precision (mAP) by averaging the AP results across all 24 fugues, and for the degraded queries, we perform an additional aggregation across the ten random seeds per noise level.

We compare twelve distances: directed Chamfer CD, trimmed Chamfer CD_ρ, directed Hausdorff HD, and percentile Hausdorff HD_ρ, with $\rho \in \{0.5, 0.6, 0.7, 0.8, 0.9\}$ (labeled 50–90). The classical directed Hausdorff distance is used as the main baseline, following the criterion underlying Romming and Selfridge-Field's pattern-matching scheme. In our implementation, nearest-neighbor distances are computed exactly, without the $(1 + \epsilon)$ approximation associated with BBD-tree nearest-neighbor search as in [10]. All distances are evaluated for $\sigma \in \{0.5, 1, 2, 4, 8, 16\}$.

4.3. Results

The results indicate a similar behavior between original, augmented and corrupted queries. Table 1 shows the mAP achieved by the distances described in Section 3.4 as a function of the temporal factor (σ, regulating the weight of the onset time dimension relative to pitch) and noise level (α). In the original condition (noise-free, $\alpha = 0$), the directed Chamfer variant performs best, achieving a peak mAP of 0.857 at $\sigma = 8$. Even with the original queries, directed Hausdorff is noticeably sensitive due to its worst-case criterion, producing an mAP ranging from 0.696 to 0.778, which is lower than the mAP of directed Chamfer, ranging between 0.836 and 0.857. Directed Hausdorff rapidly deteriorates as α increases, accentuating the difference in mAP compared to other distances. For example, at $\alpha = 0.5$, the HD range fluctuates between 0.048 and 0.123; this is to be expected, given the addition of a considerable number of outlier notes in the evaluation of the maximum nearest-neighbor distance. Trimmed Chamfer variants are much more stable, mitigating this sensitivity; at $\alpha = 0.5$, CD_{80} and CD_{70} achieve a peak mAP of 0.844 at $\sigma = 4$, while CD_{60} stays at or above 0.828 across all σ values.

Table 2 reports the results for corrupted queries, where original pitches are altered. The behavior is similar to the augmented case: trimmed Chamfer variants perform the best. For instance, CD_{90} peaks at 0.848 at $\alpha = 0.1$ and $\sigma = 4$. Directed Hausdorff again performs poorly, with an mAP at or below 0.300 for all temporal scales. As corruption becomes stronger, trimmed Chamfer remains competitive. At $\alpha = 0.4$ and at $\alpha = 0.5$, CD_{60} and CD_{50} are respectively the best performing methods for all values of σ.

4.4. Discussion

The results show that trimmed and percentile variants, such as CD_{80} and HD_{50}, can effectively filter out noise introduced by outlier notes, while pitch corruption causes a greater performance degradation, as it alters useful information in the queries. For some robust variants, non-monotonic fluctuations in mAP can also be observed for both augmented and corrupted conditions as alpha (α) increases (e.g., in Table 1, the mAP of CD_{50} at $\sigma = 0.5$: $0.821 \rightarrow 0.824$ from $\alpha = 0.4$ to $\alpha = 0.5$). However, this does not imply that increasing noise improves performance in general. Rather, it may occur since trimmed Chamfer and percentile Hausdorff methods depend only on the lowest portion (or on a low order statistic) of the ordered discrepancies between the query and candidate windows. Therefore, in original or low-noise conditions, these variants may assign a high degree of similarity to candidate windows that only partially match the query, even if these windows do not belong to the ground truth. The addition of outlier notes, however, increases the number of points the distance function must evaluate, which can make partial false matches less competitive and may lead to non-monotonic fluctuations in mAP. Similarly, for corrupted queries, pitch corruption can change which discrepancies fall within the retained subset or order statistic, producing the same effect. This issue highlights that, compared to the directed Chamfer and Hausdorff distances (whose mAP exhibits a monotonic behavior across α), partial variants are more sensitive to spurious partial matches. If the dataset contains many partial matches where the unmatched part differs significantly, this could have a negative impact on the performance of the partial variants. In the BaShoF dataset, however, this effect remains minor, suggesting that robust variants can ignore large outliers without significantly increasing partial false positives.

Directed Chamfer consistently outperforms directed Hausdorff under all conditions, and under the noise-free condition, it also matches or outperforms all the partial variants for temporal scales $\sigma \geq 2$. Overall, trimmed Chamfer performs best both at all augmentation levels and all pitch corruption levels. From a geometrical perspective, this difference reflects the contrast between a worst-case criterion and an average-based criterion. Specifically, the former is significantly inflated by any outlier notes present in the query, producing a similarity measurement that is sometimes too penalising and masks true matches. The Chamfer distance, instead, mitigates this vulnerability by averaging all nearest-neighbor distances.

5. Conclusions

In this work, we present a geometric pattern-matching approach for symbolic polyphonic music. We compare the directed Hausdorff criterion used by Romming and Selfridge-Field with the directed Chamfer, trimmed Chamfer and percentile Hausdorff distances. Experiments conducted on the BaShoF dataset [24], which we propose for

Table 1
Mean AP for original and augmented queries, averaged over 24 fugues; augmented rows are also averaged over the ten seeds.

		Chamfer						Hausdorff					
**Condition**	σ	**CD**	**CD**$_{90}$	**CD**$_{80}$	**CD**$_{70}$	**CD**$_{60}$	**CD**$_{50}$	**HD**	**HD**$_{90}$	**HD**$_{80}$	**HD**$_{70}$	**HD**$_{60}$	**HD**$_{50}$
Original ($\alpha = 0$)	0.5	0.836	**0.839**	0.837	0.830	0.804	0.767	0.696	0.761	0.777	0.812	0.799	0.766
	1	0.844	**0.846**	0.840	0.832	0.812	0.778	0.733	0.792	0.803	0.826	0.801	0.768
	2	**0.847**	0.846	0.837	0.832	0.808	0.773	0.757	0.803	0.818	0.828	0.802	0.767
	4	**0.855**	0.848	0.837	0.822	0.796	0.767	0.772	0.825	0.826	0.829	0.800	0.757
	8	**0.857**	0.844	0.830	0.819	0.795	0.767	0.778	0.829	0.835	0.826	0.798	0.757
	16	**0.853**	0.845	0.832	0.819	0.795	0.768	0.773	0.831	0.833	0.824	0.798	0.759
Augmented ($\alpha = 0.1$)	0.5	0.782	0.835	**0.836**	0.835	0.825	0.790	0.195	0.716	0.761	0.790	0.806	0.786
	1	0.815	0.844	**0.845**	0.838	0.833	0.799	0.194	0.752	0.796	0.808	0.816	0.787
	2	0.830	0.844	**0.846**	0.836	0.830	0.793	0.205	0.775	0.814	0.818	0.818	0.785
	4	0.839	**0.851**	0.847	0.832	0.818	0.786	0.222	0.805	0.826	0.828	0.821	0.778
	8	0.844	**0.850**	0.841	0.826	0.816	0.784	0.266	0.808	0.833	0.832	0.821	0.778
	16	0.842	**0.849**	0.842	0.827	0.816	0.785	0.314	0.812	0.838	0.827	0.818	0.779
Augmented ($\alpha = 0.2$)	0.5	0.737	0.828	0.832	**0.835**	0.826	0.809	0.109	0.503	0.731	0.774	0.801	0.800
	1	0.781	0.841	0.842	**0.843**	0.833	0.818	0.112	0.518	0.770	0.798	0.816	0.799
	2	0.808	0.842	**0.845**	0.842	0.831	0.814	0.115	0.546	0.793	0.816	0.818	0.803
	4	0.822	**0.850**	0.847	0.839	0.820	0.803	0.111	0.574	0.817	0.826	0.823	0.801
	8	0.829	**0.847**	0.841	0.834	0.817	0.801	0.146	0.601	0.824	0.839	0.822	0.800
	16	0.832	**0.848**	0.843	0.835	0.817	0.801	0.206	0.650	0.829	0.833	0.820	0.801
Augmented ($\alpha = 0.3$)	0.5	0.679	0.819	0.827	0.828	**0.829**	0.811	0.076	0.345	0.645	0.747	0.780	0.791
	1	0.738	0.835	**0.840**	**0.840**	0.836	0.821	0.073	0.349	0.689	0.786	0.799	0.801
	2	0.780	0.838	**0.842**	**0.842**	0.835	0.817	0.077	0.360	0.716	0.806	0.811	0.803
	4	0.806	0.844	**0.848**	0.842	0.828	0.805	0.080	0.376	0.752	0.821	0.821	0.803
	8	0.816	0.843	**0.844**	0.835	0.824	0.803	0.105	0.420	0.769	0.831	0.829	0.802
	16	0.821	0.843	**0.846**	0.837	0.825	0.803	0.150	0.481	0.789	0.834	0.826	0.803
Augmented ($\alpha = 0.4$)	0.5	0.625	0.805	0.825	0.827	**0.830**	0.821	0.060	0.245	0.506	0.723	0.773	0.801
	1	0.690	0.826	0.839	**0.840**	**0.840**	0.833	0.056	0.245	0.540	0.767	0.794	0.812
	2	0.742	0.832	0.841	**0.843**	0.839	0.830	0.057	0.247	0.561	0.791	0.808	0.815
	4	0.782	0.837	**0.847**	0.843	0.835	0.817	0.057	0.250	0.585	0.813	0.822	0.819
	8	0.798	0.835	**0.843**	0.837	0.830	0.815	0.078	0.297	0.617	0.825	0.833	0.818
	16	0.806	0.835	**0.845**	0.838	0.831	0.814	0.124	0.366	0.676	0.826	0.829	0.818
Augmented ($\alpha = 0.5$)	0.5	0.582	0.786	0.821	0.826	**0.828**	0.824	0.054	0.194	0.419	0.644	0.760	0.790
	1	0.651	0.813	0.837	0.839	**0.841**	0.836	0.048	0.197	0.432	0.699	0.788	0.801
	2	0.713	0.824	0.840	**0.841**	**0.841**	0.834	0.051	0.199	0.436	0.730	0.804	0.810
	4	0.759	0.828	**0.844**	**0.844**	0.837	0.823	0.053	0.199	0.459	0.762	0.818	0.818
	8	0.783	0.825	**0.840**	0.838	0.832	0.820	0.072	0.239	0.503	0.780	0.830	0.823
	16	0.794	0.827	**0.843**	0.840	0.832	0.820	0.123	0.309	0.580	0.795	0.827	0.822

Table 2
Mean AP for corrupted queries, averaged over 24 fugues and over the ten seeds.

		Chamfer						Hausdorff					
**Condition**	σ	**CD**	**CD**$_{90}$	**CD**$_{80}$	**CD**$_{70}$	**CD**$_{60}$	**CD**$_{50}$	**HD**	**HD**$_{90}$	**HD**$_{80}$	**HD**$_{70}$	**HD**$_{60}$	**HD**$_{50}$
Corrupted ($\alpha = 0.1$)	0.5	0.763	0.832	0.831	**0.834**	0.811	0.785	0.182	0.698	0.752	0.784	0.793	0.780
	1	0.800	**0.843**	0.841	0.841	0.818	0.793	0.184	0.738	0.790	0.805	0.802	0.782
	2	0.819	**0.843**	0.842	0.840	0.815	0.788	0.191	0.763	0.804	0.818	0.803	0.783
	4	0.830	**0.848**	0.841	0.831	0.803	0.781	0.209	0.791	0.818	0.825	0.804	0.774
	8	0.835	**0.845**	0.834	0.828	0.801	0.780	0.252	0.802	0.828	0.830	0.803	0.775
	16	0.834	**0.846**	0.836	0.829	0.801	0.779	0.300	0.806	0.830	0.828	0.802	0.776
Corrupted ($\alpha = 0.2$)	0.5	0.673	0.819	0.826	**0.828**	0.819	0.796	0.096	0.432	0.689	0.750	0.785	0.786
	1	0.727	0.834	0.838	**0.840**	0.828	0.805	0.095	0.441	0.732	0.783	0.797	0.791
	2	0.763	0.836	**0.840**	**0.840**	0.827	0.802	0.096	0.459	0.761	0.802	0.804	0.792
	4	0.788	0.840	**0.842**	0.835	0.817	0.792	0.100	0.485	0.788	0.814	0.808	0.785
	8	0.798	0.836	**0.837**	0.831	0.814	0.791	0.128	0.517	0.803	0.825	0.813	0.785
	16	0.803	0.833	**0.838**	0.831	0.813	0.790	0.183	0.557	0.810	0.824	0.810	0.785
Corrupted ($\alpha = 0.3$)	0.5	0.546	0.773	0.804	**0.814**	0.808	0.799	0.063	0.237	0.448	0.682	0.744	0.774
	1	0.608	0.805	0.825	**0.830**	0.823	0.813	0.063	0.237	0.478	0.727	0.766	0.786
	2	0.657	0.816	0.830	**0.834**	0.826	0.811	0.061	0.243	0.510	0.758	0.783	0.790
	4	0.705	0.818	**0.832**	**0.832**	0.817	0.801	0.070	0.253	0.524	0.783	0.796	0.786
	8	0.738	0.815	**0.828**	**0.828**	0.813	0.799	0.087	0.287	0.557	0.802	0.804	0.790
	16	0.751	0.809	**0.829**	0.828	0.812	0.798	0.133	0.334	0.617	0.804	0.803	0.790
Corrupted ($\alpha = 0.4$)	0.5	0.406	0.672	0.751	0.781	**0.801**	0.795	0.042	0.130	0.253	0.422	0.673	0.743
	1	0.456	0.713	0.784	0.809	**0.822**	0.815	0.044	0.134	0.248	0.459	0.712	0.762
	2	0.510	0.736	0.797	0.816	**0.826**	0.817	0.045	0.129	0.253	0.479	0.742	0.772
	4	0.570	0.744	0.798	0.812	**0.822**	0.809	0.050	0.131	0.263	0.491	0.765	0.778
	8	0.626	0.745	0.790	0.806	**0.818**	0.807	0.066	0.161	0.292	0.536	0.785	0.785
	16	0.662	0.743	0.784	0.801	**0.817**	0.806	0.110	0.209	0.357	0.581	0.788	0.786
Corrupted ($\alpha = 0.5$)	0.5	0.297	0.555	0.668	0.726	0.762	**0.776**	0.036	0.082	0.162	0.275	0.455	0.662
	1	0.336	0.599	0.708	0.768	0.798	**0.803**	0.036	0.087	0.156	0.288	0.502	0.691
	2	0.374	0.625	0.726	0.782	0.808	**0.810**	0.039	0.085	0.145	0.293	0.545	0.712
	4	0.435	0.640	0.730	0.780	0.804	**0.805**	0.046	0.094	0.156	0.295	0.544	0.733
	8	0.499	0.649	0.725	0.771	0.801	**0.804**	0.060	0.113	0.181	0.331	0.587	0.750
	16	0.556	0.650	0.714	0.763	0.800	**0.802**	0.104	0.152	0.239	0.390	0.619	0.754

symbolic polyphonic pattern-matching evaluation, demonstrate that these alternatives outperform the directed Hausdorff distance in both noise-free and noisy conditions. Specifically, directed Chamfer performs best on noise-free queries, while trimmed Chamfer is more effective against noise caused by both added outlier notes and pitch corruption. Future work includes reducing runtime through a hybrid approach inspired by recent advances in pattern discovery [29], using deep neural networks as semantic candidate pre-filters alongside the proposed distances.

CRediT authorship contribution statement

Antonio Rizzitiello: Writing – original draft, Investigation, Conceptualization, Methodology, Resources, Software, Formal analysis, Validation, Data curation. **Riccardo Giampiccolo:** Writing – review & editing, Formal analysis, Methodology, Supervision, Project administration. **Achille Frigeri:** Writing – review & editing, Supervision. **Augusto Sarti:** Writing – review & editing, Supervision, Project administration. **Alberto Bernardini:** Writing – review & editing, Supervision, Methodology, Project administration.

Declaration of competing interest

The authors declare that they have no known competing financial interests or personal relationships that could have appeared to influence the work reported in this paper.

Data availability

The GitHub repository associated with this study is available at https://github.com/polimi-ispl/sm_geompm.

References

[1] M. Schedl, E. Gómez, J. Urbano, Music information retrieval: Recent developments and applications, Found. Trends Inf. Retr. 8 (2014) 127–261. doi:10.1561/1500000042.

[2] A. Sarti, F. Antonacci, A. Bernardini, Music representation and modelling: A signal processing perspective, in: C. Johnson, A. King (Eds.), Music, Technology, Innovation: Industry and Educational Perspectives, Routledge, 2024, pp. 154–170. doi:10.4324/9781003041474-11.

[3] J. C. Bartlett, W. J. Dowling, Recognition of transposed melodies: A key-distance effect in developmental perspective, J. Exp. Psychol. Hum. Percept. Perform. 6 (1980) 501–515.

[4] R. Timmers, R. Ashley, Emotional ornamentation in performances of a Handel sonata, Music Percept. 25 (2007) 117–134. doi:10.1525/mp.2007.25.2.117.

[5] B. H. Repp, Obligatory "expectations" of expressive timing induced by perception of musical structure, Psychol. Res. 61 (1998) 33–43. doi:10.1007/s004260050011.

[6] E. Ukkonen, K. Lemström, V. Mäkinen, Geometric algorithms for transposition invariant content-based music retrieval, in: Proc. 4th Int. Conf. Music Inf. Retr. (ISMIR), 2003, pp. 193–199. doi:10.5281/zenodo.1417477.

[7] A. Lubiw, L. Tanur, Pattern matching in polyphonic music as a weighted geometric translation problem, in: Proc. 5th Int. Conf. Music Inf. Retr. (ISMIR), 2004, pp. 289–296.

[8] P. Di Lorenzo, G. Di Maio, The Hausdorff metric in the melody space: A new approach to melodic similarity, in: Proc. 9th Int. Conf. Music Percept. Cogn. (ICMPC), 2006, pp. 1037–1043.

[9] R. Typke, Music Retrieval based on Melodic Similarity, Ph.D. thesis, Utrecht Univ., 2007.

[10] C. A. Romming, E. Selfridge-Field, Algorithms for polyphonic music retrieval: The Hausdorff metric and geometric hashing, in: Proc. 8th Int. Conf. Music Inf. Retr. (ISMIR), 2007, pp. 457–462.

[11] K. Lemström, String Matching Techniques for Music Retrieval, Ph.D. thesis, Univ. Helsinki, Dept. Comput. Sci., 2000. Report A-2000-4.

[12] V. Mäkinen, G. Navarro, E. Ukkonen, Algorithms for transposition invariant string matching, in: Proc. 20th Annu. Symp. Theor. Aspects Comput. Sci. (STACS), volume 2607 of *Lect. Notes Comput. Sci.*, 2003, pp. 191–202. doi:10.1007/3-540-36494-3_18.

[13] M. Mongeau, D. Sankoff, Comparison of musical sequences, Comput. Humanit. 24 (1990) 161–175. doi:10.1007/BF00117340.

[14] J. S. Downie, Evaluating a Simple Approach to Music Information Retrieval: Conceiving Melodic N-Grams as Text, Ph.D. thesis, Univ. Western Ontario, 1999.

[15] S. Doraisamy, S. M. Rüger, Robust polyphonic music retrieval with N-grams, J. Intell. Inf. Syst. 21 (2003) 53–70.

[16] D. Rizo, Symbolic Music Comparison with Tree Data Structures, Ph.D. thesis, Univ. Alicante, 2010.

[17] A. Pinto, R. H. van Leuken, M. F. Demirci, F. Wiering, R. C. Veltkamp, Indexing music collections through graph spectra, in: Proc. 8th Int. Conf. Music Inf. Retr. (ISMIR), 2007, pp. 153–156.

[18] H. H. Hoos, K. Renz, M. Görg, GUIDO/MIR — an experimental musical information retrieval system based on GUIDO music notation, in: Proc. 2nd Int. Symp. Music Inf. Retr. (ISMIR), 2001, pp. 41–50.

[19] T. Cheng, S. Fukayama, M. Goto, Comparing RNN parameters for melodic similarity, in: Proc. 19th Int. Soc. Music Inf. Retr. Conf. (ISMIR), 2018, pp. 763–770. doi:10.5281/zenodo.1492529.

[20] F. Karsdorp, P. van Kranenburg, E. Manjavacas, Learning similarity metrics for melody retrieval, in: Proc. 20th Int. Soc. Music Inf. Retr. Conf. (ISMIR), 2019, pp. 478–485.

[21] F. Hausdorff, Grundzüge der Mengenlehre, Veit & Comp., 1914.

[22] H. G. Barrow, J. M. Tenenbaum, R. C. Bolles, H. C. Wolf, Parametric correspondence and Chamfer matching: Two new techniques for image matching, in: Proc. 5th Int. Joint Conf. Artif. Intell. (IJCAI), 1977, pp. 659–663.

[23] D. P. Huttenlocher, G. A. Klanderman, W. J. Rucklidge, Comparing images using the Hausdorff distance, IEEE Trans. Pattern Anal. Mach. Intell. 15 (1993) 850–863. doi:10.1109/34.232073.

[24] A. Rizzitiello, R. Giampiccolo, A. Sarti, A. Bernardini, BaShoF: A symbolic dataset of Bach and Shostakovich fugues for polyphonic pattern matching with original, augmented and corrupted queries, 2026. Manuscript co-submitted to Data in Brief.

[25] K. Lemström, A. Pienimäki, On comparing edit distance and geometric frameworks in content-based retrieval of symbolically encoded polyphonic music, Musicae Sci. 11 (2007) 135–152.

[26] K. Lemström, A. Pienimäki, Approaches for content-based retrieval of symbolically encoded polyphonic music, in: Proc. 9th Int. Conf. Music Percept. Cogn. (ICMPC), 2006, pp. 1028–1035.

[27] D. Yang, T. J. Tsai, Piano sheet music identification using dynamic N-gram fingerprinting, Trans. Int. Soc. Music Inf. Retr. 4 (2021) 42–51. doi:10.5334/tismir.70.

[28] D. Malandrino, R. De Prisco, M. Ianulardo, R. Zaccagnino, An adaptive meta-heuristic for music plagiarism detection based on text similarity and clustering, Data Min. Knowl. Discov. 36 (2022) 1301–1334. doi:10.1007/s10618-022-00835-2.

[29] J.-Y. Wang, Y.-C. Kuo, L. Su, Improving motif discovery of symbolic polyphonic music with motif note identification, Trans. Int. Soc. Music Inf. Retr. 8 (2025) 353–371. doi:10.5334/tismir.250.

[30] Z. Yin, F. Reuben, S. Stepney, T. Collins, Deep learning's shallow gains: A comparative evaluation of algorithms for automatic music generation, Mach. Learn. 112 (2023) 1785–1822.

[31] S. Bruhn, J. S. Bach's Well-Tempered Clavier: In-depth Analysis and Interpretation, Mainer International Ltd., 1993.

[32] M. Giraud, R. Groult, E. Leguy, F. Levé, Computational fugue analysis, Comput. Music J. 39 (2015) 77–96. doi:10.1162/COMJ_a_00300.
